# Empirical model for short-time prediction of COVID-19 spreading

**DOI:** 10.1371/journal.pcbi.1008431

**Published:** 2020-12-09

**Authors:** Martí Català, Sergio Alonso, Enrique Alvarez-Lacalle, Daniel López, Pere-Joan Cardona, Clara Prats

**Affiliations:** 1 Comparative Medicine and Bioimage Centre of Catalonia (CMCiB), Fundació Institut d’Investigació en Ciències de la Salut Germans Trias i Pujol, Badalona, Catalonia, Spain; 2 Department of Physics, Universitat Politècnica de Catalunya (UPC-BarcelonaTech), Barcelona, Catalonia, Spain; 3 Experimental Tuberculosis Unit, Fundació Institut d’Investigació en Ciències de la Salut Germans Trias i Pujol, Universitat Autònoma de Barcelona, Badalona, Catalonia, Spain; 4 Centro de Investigación Biomédica en Red de Enfermedades Respiratorias, Madrid, Spain; Institute for Disease Modeling, UNITED STATES

## Abstract

The appearance and fast spreading of Covid-19 took the international community by surprise. Collaboration between researchers, public health workers, and politicians has been established to deal with the epidemic. One important contribution from researchers in epidemiology is the analysis of trends so that both the current state and short-term future trends can be carefully evaluated. Gompertz model has been shown to correctly describe the dynamics of cumulative confirmed cases, since it is characterized by a decrease in growth rate showing the effect of control measures. Thus, it provides a way to systematically quantify the Covid-19 spreading velocity and it allows short-term predictions and longer-term estimations. This model has been employed to fit the cumulative cases of Covid-19 from several European countries. Results show that there are systematic differences in spreading velocity among countries. The model predictions provide a reliable picture of the short-term evolution in countries that are in the initial stages of the Covid-19 outbreak, and may permit researchers to uncover some characteristics of the long-term evolution. These predictions can also be generalized to calculate short-term hospital and intensive care units (ICU) requirements.

## Author summary

Covid-19 has brought the international scientific community into the eye of a storm. Collaboration between researchers, public health workers, and politicians is essential to deal with this challenge. One of the pieces of the puzzle is analysis of epidemiological trends so that both the current and immediate future situation can be carefully evaluated. For this reason we have employed a daily generic growing function to describe the cumulative cases of Covid-19 in several countries and regions around the world, and particularly the European countries during the Covid-19 outbreak. Our model is completely empirical, meaning it relies solely on the daily data update of new cases and does not require assumptions to make predictions. In this manuscript, we detail the methods employed and the degree of confidence we have obtained during this process. We obtain predictions with a success greater than 90%, which means that around 90% of the value of the reported cases are inside the prediction intervals. This can be used for other researchers collaborating with and advising health institutions around the world during the Covid-19 outbreak or any other epidemic that follows the same pattern. We hope it may help facilitate policy decisions, the review of in-place confinement measures, and the development of new protocols.

## Introduction

A disease outbreak is always a challenge for public health control systems. When the outbreak is caused by a new agent able to cause a pandemic, the challenge is even greater and should involve the whole research community as well. Globalization plays a double role in this context; on the one hand, it increases the risk of the outbreak evolving towards a pandemic, while on the other, the sharing of data and strategies increases the likelihood of controling it. The new SARS-CoV-2 virus (severe acute respiratory syndrome coronavirus 2) has put the international community at the brink of a global disaster. National and local governments are working with public health agencies hand in hand to slow down, and eventually control, the spread of Covid-19 [1].

Daily availability of data about confirmed cases of Covid-19 in different regions is a unique opportunity for basic scientists to contribute to its control by carefully analyzing trends. In particular, mathematical models are widespread, as are consolidated tools to extract valuable information from the reported data on Covid-19 and help making predictions [2]. Classic SIR and SEIR models (i.e., compartment models that divide a population into Susceptible, Exposed, Infectious and Recovered) are being currently employed to evaluate and predict the spreading of the epidemic episodes [3]. They were employed in the description of the Ebola epidemic in 1995 [4] and 2014 [5] and in the more recent SARS epidemic of 2003 [6], among others. After the SARS epidemic in 2003, in order to account for the control efforts of governments, some modifications were introduced into the SEIR model to evaluate control measures [7, 8]. Furthermore, the analysis of SEIR models has been used for the modeling of Covid-19’s spread in China in a effort to fit the characteristic values [9, 10]. However, during the development of the epidemic, government measures are the key drivers of the epidemic. The evolution of the disease is completely different depending on the strength and type of restrictions on mobility and social life that governments implement. The evolution of the disease in a situation where there is a total lockdown is very different from a situation where only specific restrictions to mobility apply, such as forbidding large gatherings. Similarly, the evolution is different depending on the nature of the policy initiatives. Closure of schools or bars affects the evolution differently than closure of nightlife venues. Simple SIR and SEIR models are not designed to deal with this type of situation where the network of contacts and its changes due to policy are key. SIR and SEIR models deal properly with epidemics where the key element of the evolution is the the total number of susceptible population. Its reduction, as the epidemic advances, gives the characteristic peak-like evolution. In the case of Covid-19, the total number of susceptible is not important because cumulative cases in the countries are far from achieving herd immunity [11].

There is, however, another approach based on the phenomenological comparison of the curve of cumulative cases with a typical function for growing processes. Evaluating the curve during a window of days before a particular day *t* allows prediction of the future short-time behavior tendency at time *t* + Δ*t* [12]. In fact, the use of a growing function has some important advantages. Typically, the first growing function chosen is the Verhulst equation [13] which is the solution of the logistic population model and its generalization [14, 15], or the Richards model [16], which has been employed in several epidemics of smallpox, influenza, and Ebola, among others [14]. Some of these dynamic phenomenological growth models to study epidemic outbreaks have been compared in the initial phases of the Covid-19 epidemic for short-term forecasting [17].

A similar growth model is the Gompertz function [18] where the main difference is the replacement of the saturation of the growing factor, linear for the Verhulst equation and non-linear for the Richards, and generalized Verhulst model, by an exponential decrease. These functions are similar and they have been used in the description of epidemics and in particular for studying different epidemic episodes [19, 20]. While the logistic equation produces a symmetric bell-shaped function for new cases, the Gompertz model gives rise to an asymmetric function with fast growth of new cases combined with a slow decrease, which is closer to the distribution of new cases observed in different countries during some epidemics. In this manuscript we demonstrate that the asymmetric nature of the Gompertz model is the proper framework to study epidemics in which control measures are at the heart of the evolution, since it captures the dynamic nature of the variation due to social distance measures.

Here, we employ the Gompertz growing function to analyze the dynamics of the spreading of Covid-19 in 28 European countries to make short-time predictions of the new cases for successive days. We forecast the dynamics of the pandemic in a similar fashion to the forecasting done previously with the Verhulst equation and the Richards model for Ebola epidemics [21]. The methodology and the results discussed here were employed for the writing of daily reports [22] at the very beginning of the epidemics. Later on, similar methodologies were employed to fit worldwide data [23, 24], and the data in particular countries like Mexico [25] and Brazil [26], among others. We have also applied similar methodology for the prediction of cases for hospitals and intensive care units (ICUs).

It is important to note that we forecast the dynamics of the pandemic using a phenomenological model, obtaining short-term predictions for daily new cases with over 90 percent success (see below). These data may be useful for public health policy makers and they are easily reproducible by scientists all over the world.

## Materials and methods

After a short note about data acquisition, we describe the function employed for the fitting of the data and then describe the evaluation of the errors associated with these calculations.

### Data acquisition

All the data employed in this manuscript have been downloaded from public repositories of the European Centre for Disease Prevention and Control (ECDC). The data contain the daily list of cumulative cases for all the countries of the world reporting the data and it is a fully open source [27], originally from [28]. Similar data are supplied by the World Health Organization (WHO) [29].

### Short review of Gompertz equation

**Fig 1:**
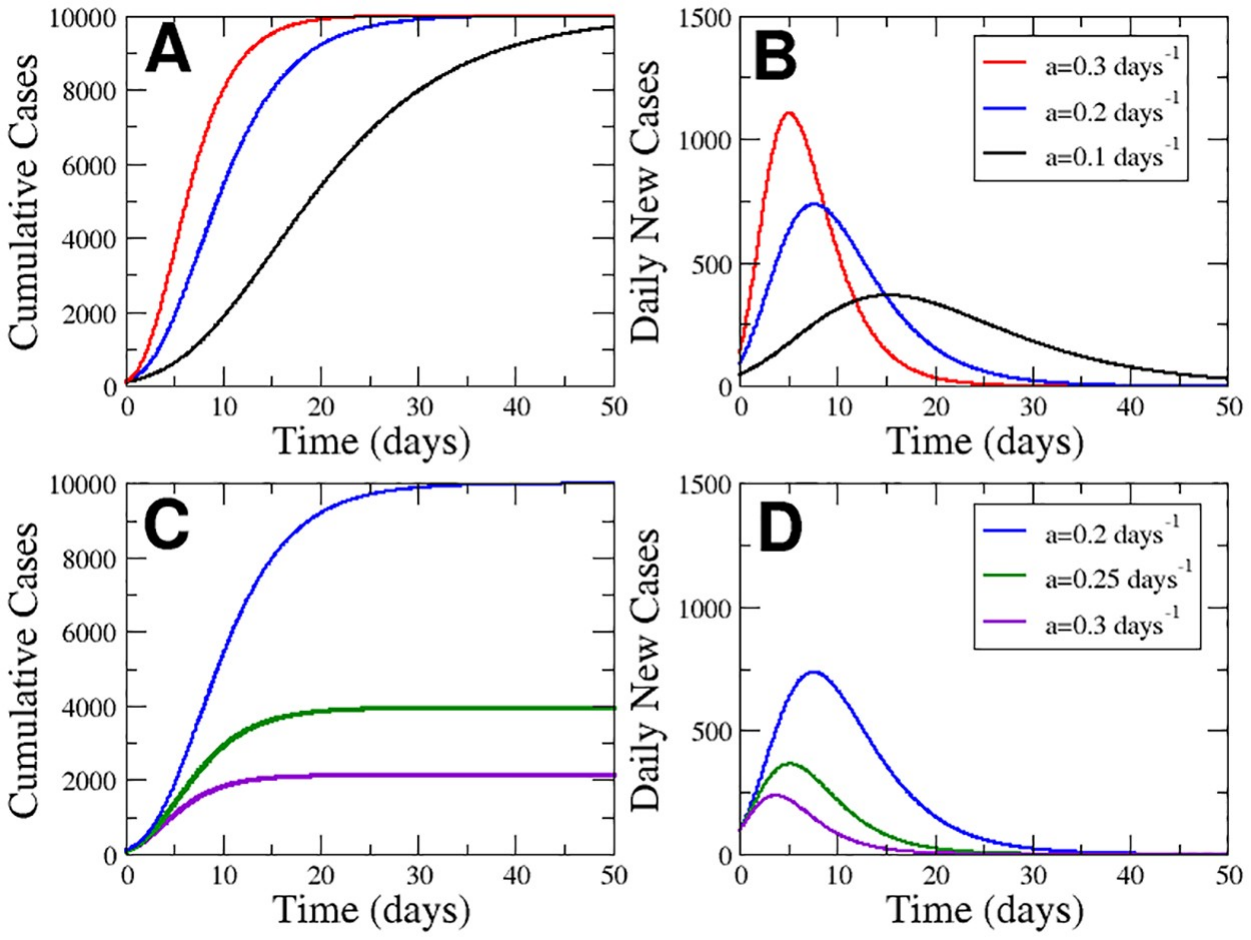
Properties of Gompertz function. Evolution of the cumulative cases (A) and new cases (B) keeping *K* = 10^4^ for three different values of a. Evolution of cumulative cases (C) and new cases (D) keeping *μ*_0_ = 0.92 for three different values of *a*.

We employ the Gompertz model for growing processes to model the cumulative cases of Covid-19. The equation was originally proposed as a means to explain human mortality curves [18], and it has been further employed in the description of growth processes, for example, bacterial colonies [30] and tumors [31]. The Gompertz equation reads:

$$\begin{array}{c} N\left(t\right)=Ke^{-\ln\left(\frac{K}{N_{0}}\right)e^{-at}} \end{array}$$ (1)

where the parameter *K* corresponds to the final number of cases, *N*_0_ is the initial number of cases for the definition of the origin of time, and parameter *a* is the rate of decrease in the initially exponential growth; see curves in Fig 1A for different values of *a*. For the beginning of the epidemic, corresponding to *t* → 0, the Eq (1) reduces to an initial exponential growth $N=N_{0}e^{\mu_{0}t}$ with rate *μ*_0_ = *a* ln (*K*/*N*_0_). After time *t*_*p*_ the growth flattens asymptotically to the final value given by the saturation parameter *K*. To compare with the cumulative cases of Covid-19 we begin to measure above 100 cases (*N*_0_ = 100). The exponential rate *μ*_0_ provides us with the relation between the parameters *K* and *a*.

In addition, the Gompetz function can be interpreted as the solution for the next couple of ordinary differential equations:

$$\begin{array}{c} \frac{dN}{dt}=\mu N,\;\;\;\;\;\;\;\;\;\;\;\;\frac{d\mu }{dt}=-a\mu ; \end{array}$$ (2)

which corresponds, respectively, to an exponential growth with a growing rate *μ* which exponentially decreases with rate *a*.

The Gompertz function shows the cumulative cases. Therefore the temporal derivative of the cumulative cases is basically the new cases. Performing the temporal derivative we obtain:

$$\begin{array}{c} N_{n}=\frac{dN}{dt}=aKe^{-\ln(\frac{K}{N_{0}})e^{-at}}(\ln(\frac{K}{N_{0}})e^{-at}); \end{array}$$ (3)

the dynamics of which as as function of time are plotted in Fig 1B.

Fixing the total values of cases (*K* = 10^4^) we can study the effect of a rapid decay of the growing rate, related to a large value for *a* with a slower decrease, determined by a low value for *a*. See Fig 1A for a visual inspection of the effect of this parameter *a*. The increase in the parameter *a* produces a delay in the growing process and delay of the peak, see Fig 1B, where the area of the curve is constant because of the conservation of the final value *K*. However, in Fig 1C we fix the initial exponential growth determined by *μ*_0_ and increase the parameter *a*, which decreases the final value of total cases. The amplitude of the peak is decreased by the increase in the rate *a* when the initial growth is fixed, see Fig 1D.

We see a maximum of new cases in Fig 1, for which the inflection point (*t*_*p*_) can be calculated:

$$\begin{array}{c} t_{p}=\frac{1}{a}\ln(\ln(\frac{K}{N_{0}}))=\frac{1}{a}\ln(\frac{\mu_{0}}{a}); \end{array}$$ (4)

and we can also estimate the time necessary to arrive at 90% of the total value of cases *K*:

$$\begin{array}{c} t_{90}=\frac{-1}{a}\ln(\frac{-\ln0.9}{\ln(K/N_{0})}). \end{array}$$ (5)

The last two expresions clearly mark the effect of the parameter *a*. The larger the value of *a*, the faster the appearance of the peak and the arrival at 90% of cases, see Fig 1B and 1D.

### Evaluation and propagation of errors

The fitting of the Gompertz function to the data is done with a matlab routine using the minimum least squares method [32]. This method allows for evaluation of the set of model parameters that provide the best fit for the Gompertz model to the data. Furthermore, the method also provides the error associated with the values of the fitting constants. The performance of the fitting can be evaluated with the statistical parameter *R*^2^, available from the procedure of the calculation of the fitting.

We employ the explicit values of the fitted parameters to make our predictions. The propagation of the uncertainty or error in the calculation of the predictions can be done using the classical methods of propagation of errors [32]. In short, if we have a quantity *U* which depends on two magnitudes *U* = *U*(*a*, *b*) and these magnitudes have their uncertainties *a* ± *δa* and *b* ± *δb*, if we assume the quantities are uncorrelated we can calculate the uncertainty of the new quantity as:

$$\begin{array}{c} \delta U=\sqrt{{\left(\frac{\partial U}{\partial a}\right)}^{2}\delta a^{2}+{\left(\frac{\partial U}{\partial b}\right)}^{2}\delta b^{2}}, \end{array}$$ (6)

expression which is employed for example for the calculation of the time to peak; see Eq (4) and for the calculation of the time to reach the 90% of the expected value of *K*. For example, we calculate the dependence of the error in *t*_*p*_ on the parameters *a* and *K*, see Eq (4):

$$\begin{array}{c} \delta t_{p}=\sqrt{{\left(\frac{ln(\ln(\frac{K}{N_{0}}))}{a^{2}}\right)}^{2}\delta a^{2}+{\left(\frac{K}{aln(\frac{K}{N_{0}})}\right)}^{2}\delta K^{2}}; \end{array}$$ (7)

a similar calculation can be made for the error of *t*_90_, see Eq (5).

## Results

We make some predictions using the Gompertz function to fit the cumulative cases of Covid-19 in different countries where the epidemic was developed enough in April, 2020. Next, we show such predictions and the main applications of the Gompertz model for the characterization of the epidemic.

### Gompertz model fits the number of cases for recovered regions

**Fig 2:**
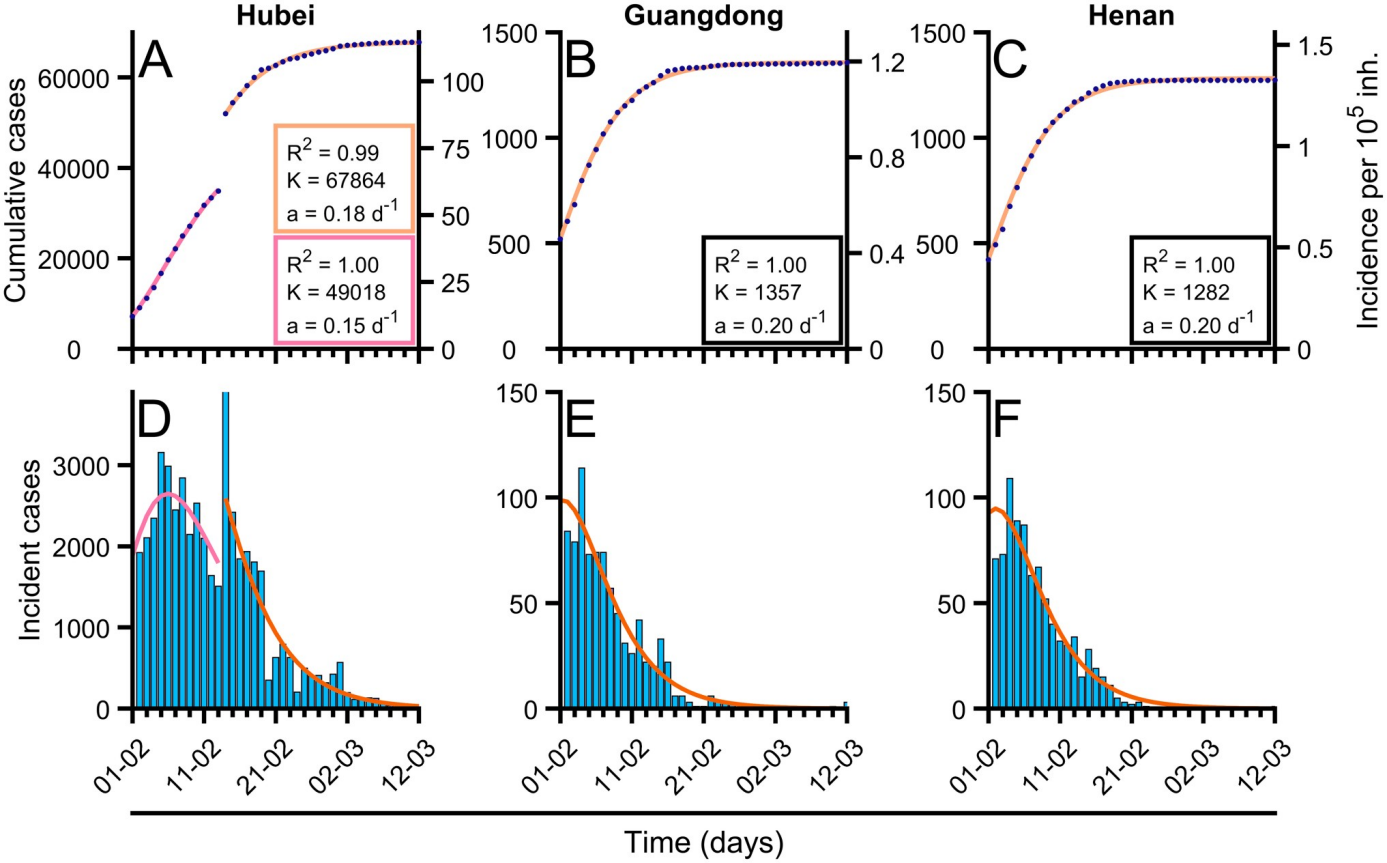
Fitting of Gompertz function to the cumulative confirmed cases of Covid-19 in different countries. Fitting of Gompertz function to the cumulative confirmed cases of Covid-19 in different countries. (A-C) Evolution of total confirmed cases in different regions of China (blue dots) and fitted Gompertz function in each region (orange solid line). (D-F) Evolution of new cases in different regions of China (blue bars) and fitted Gompertz function in each region (orange solid line), with *R*^2^ = 0.65, *R*^2^ = 0.72 (Hubei), *R*^2^ = 0.94 (Guangdong), *R*^2^ = 0.94 (Henan). In the case of Hubei (A, D), as there was a sudden change in reporting criterion there were two fitted Gompertz adjustments: pre-change (pink solid line) and post-change (solid orange line). The obtained values of parameter *a* (related with growth rate), *K* (final number of cases), and mean-squared error (*R*^2^) are shown for each of the fittings. Data were updated on March 5, 2020 from [29].

Gompertz model [33] correctly describes the trend of the cumulative confirmed cases as seen in Fig 2 where the values of the statistical measure *R*^2^ are close to 1. We perform a systematic analysis of the dynamics of the cumulative cases of Covid-19 in different regions in China where the spreading of the epidemic finished; see for example the three regions shown in Fig 2 where the Gompertz function has been fitted. Note, however, that the fit in Hubei is divided into two regions because of a change in the protocol for reporting cases. The new cases are also fitted with relatively large *R*^2^ values; see three panels below in Fig 2, with the function derived from the Gompertz model; see Eq (3). To fit the Gompertz function to the data we obtain the values of the fitting parameters *a* and *K*, which accompany the corresponding panels in Fig 2.

**Fig 3:**
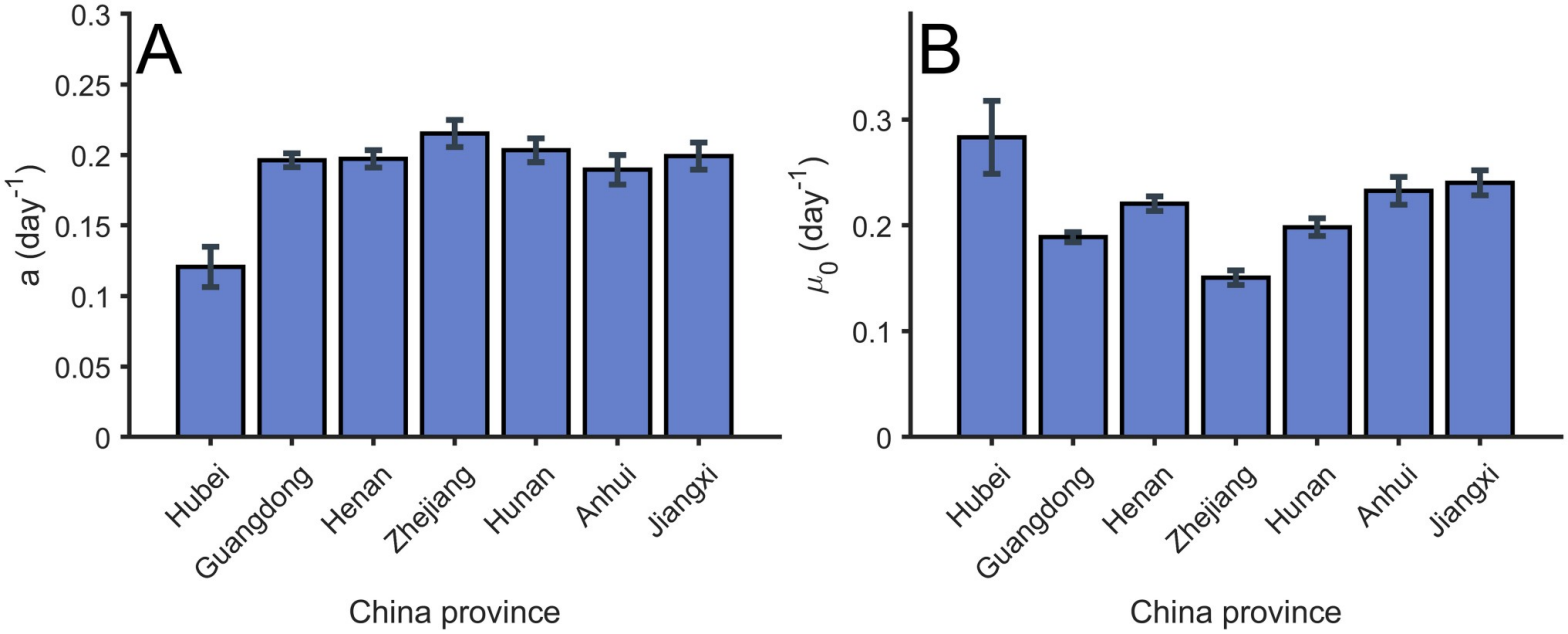
Values of parameter *a* and *μ*_0_ for the Gompertz function in different regions in China. (A) Value of parameter *a* obtained from the fitting of the total confirmed cases. (B) Value of parameter *μ*_0_ obtained from the fitting of the total confirmed cases. Error bars parameters with confidence intervals of level *α* = 0.01.

Let us focus now on this classification according to control measures. We show in Fig 3 the values resulting for the fitting of the Gompertz function to the data from several regions in China. Assuming that the measures of control taken in China were considered very restrictive, we can assume that the values obtained in these regions, and shown in Fig 3A, are the upper limit of the parameter for other countries. The actual value obtained is around a = 0.2 days^−1^.

Furthermore, we can evaluate the value of parameter *μ*_0_ for the initial exponential growth of the different regions; see details in Fig 3B. We obtain similar quantities in all the regions in China and it provides information about the growing rate of the epidemic in China, the value of which is, in these cases, similar to the decreasing rate *a* calculated above.

### Short-term predictions obtained from Gompertz model

Although understanding of the epidemic from the final picture of the dynamics is a valuable result for the treatment of future epidemics, the main goal of the modeling of epidemics is the actual possibility of prediction of the behavior during the incidence of the epidemic. We have used the Gompertz model during the epidemic episode of Covid-19 in several countries in Europe.

**Fig 4:**
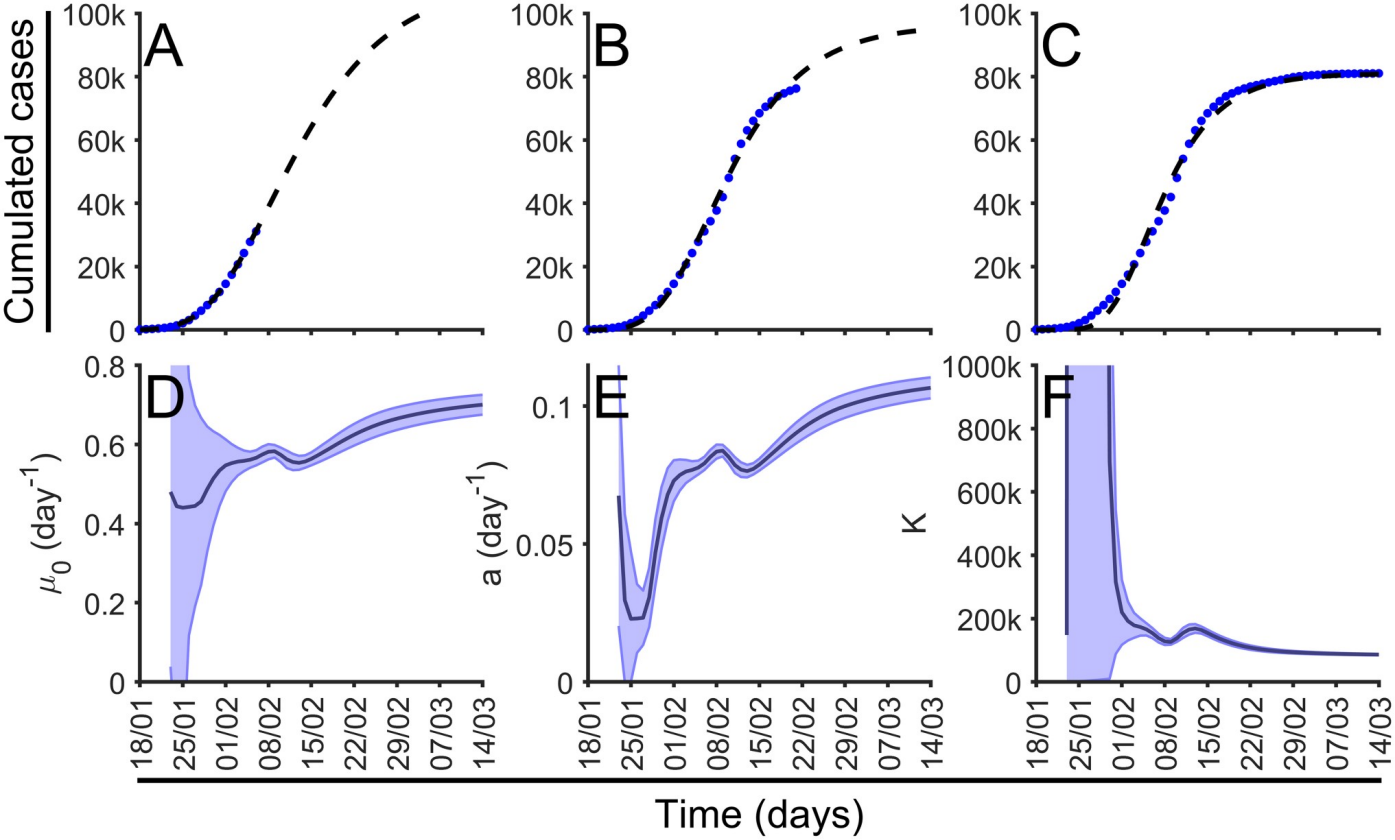
Dynamical fitting of Gompertz function and parameters evolution. (A-C) Gompertz fitting for China at three different time points, 7 February, 20 February and 14 March. Number of cumulative cases (blue dots) shown together with the function fitted (black dash line). (D-F) Dynamic calculation of parameters *μ*_0_, *a*, and *K* in dark blue; light blue mark error bars parameters with confidence intervals of level *α* = 0.01.

First, we evaluated the predictions with the data obtained in the different regions in China to estimate the error of the fitting procedure of the Gompertz function before saturation of the number of cases. We began with the first day after 100 cumulative cases of Covid-19 and we successively fit a Gompertz function to the previous values of cumulative cases to estimate the values of parameters *a* and *μ* which permits estimation of the values for the cases for the next days. In Fig 4A, 4B and 4C), we show the fitting of the Gompertz function to the values of cumulative cases at three different times. The fittings of the function at different times differ with the final values of the total function shown in Fig 2D and 2E and therefore the values of the three fittings produce different values of parameters *a* and *μ*_0_. However, the evolution of the values converges to the global fitting of the function to the whole set of data, see Fig 3D, 3E and 3F.

Such large variations on the parameter fittings show clearly that long-time predictions are complicated. However, we can perform short-time predictions for the number of new cases if we extrapolate the Gompertz function to the near future with the updated values of *a* and *μ*_0_ for the cumulative cases. We systematically extrapolated the new cases for each temporal data of the series of cumulative cases of Covid-19 in the different regions in China and obtained a successful agreement of the predictions with the actual data for the whole series; see below for more extensive results taking into account a larger number of countries.

### Short-term predictions can be applied to ongoing epidemics

**Fig 5:**
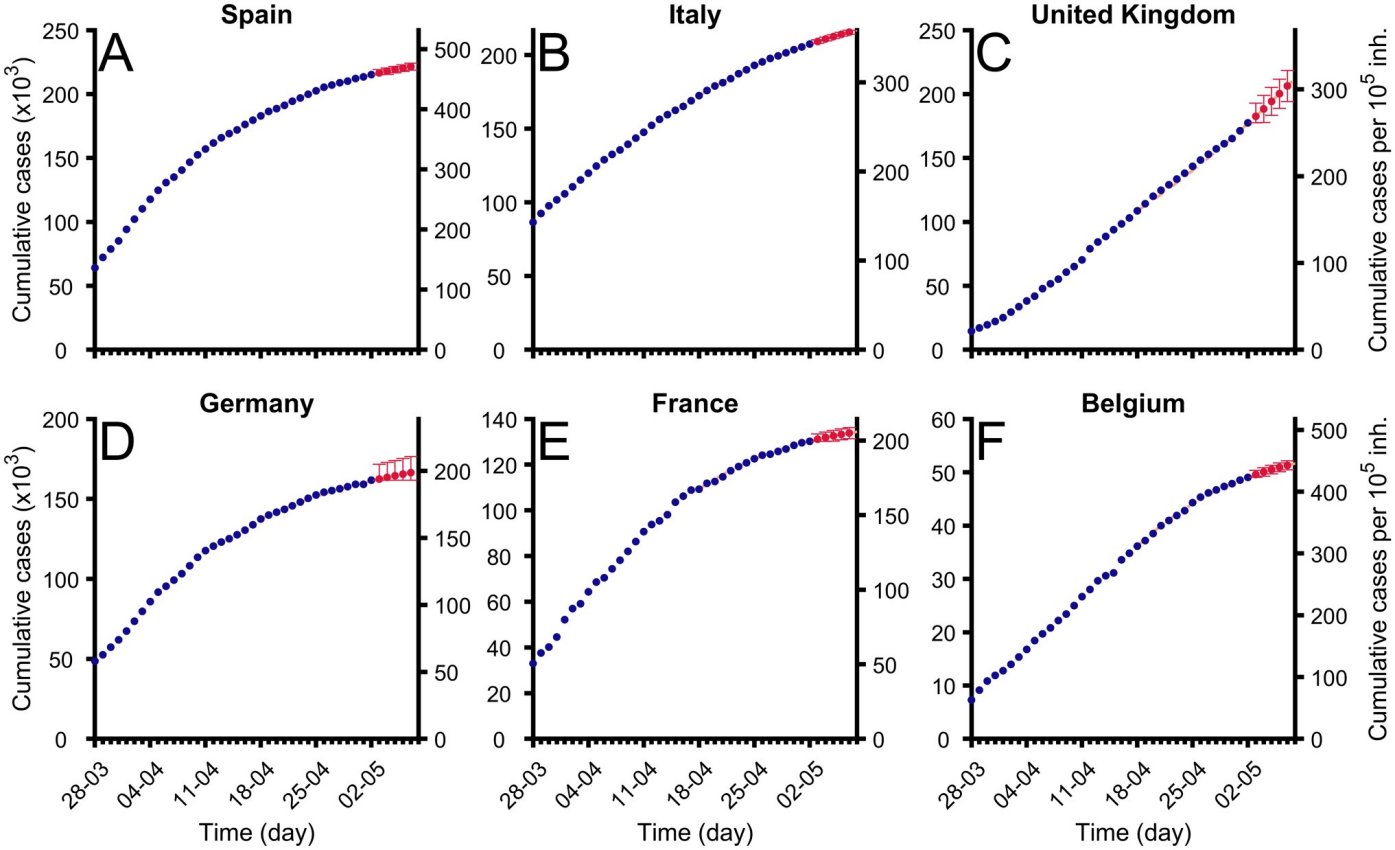
Fitting of Gompertz function to cumulative cases in some countries in Europe. Evolution of total confirmed cases in different regions (blue dots) and fitted Gompertz function in each region (orange dashed line). Red points show predictions for next 5 days and error bars marks their confidence interval levels *α* = 0.01. Data were updated on April 9, 2020 from [29]. (A) Spain (B) Italy (C) Germany (D) France (E) United Kingdom, and (F) Belgium.

The epidemic is still spreading throughout Europe and we have been fitting the Gompertz function to the total cumulative cases for two months (March and April 2020). Most of the countries had already arrived at the saturation stage and the fitting of the function allows evaluation of the control measures. See the examples in Fig 5, where a Gompertz function satisfactorily fits the existing data. Note that Gompertz function is able to fit countries at different epidemiological phases. We systematically assessed short-time predictions for all European countries, the United Kingdom, Norway, and Switzerland every day from March 17th [34], as well as for Spanish and Italian regions [35].

Typically, the evolution of confirmed cases shows a biphasic behavior: an initial lag phase where no significant increase in the incidence is observed, which would correspond to the period where most of the cases are imported, followed by a subsequent phase where growth is evident, which would be a reflection of triggering local transmission. Gompertz model is fitted to the later phase, i.e., it is applied from the moment when a clear increase in confirmed cases is observed, typically above 100 cases to avoid the evaluation of the beginning of the epidemic dominated by the importing of cases from other zones.

As an example of these predictions, we refer the reader to Table 1. The cases correspond to the evolution of the values of the cumulative cases up to April 29, 2020. We show the predictions for some countries in Europe of the algorithm based on the Gompertz function, for the next 1, 3, and 5 days. The rate of success in this example is representative for the algorithm; see following section.

**Table 1:** Short-term predictions on April 29, 2020 with Gompertz model. Countries were sorted by number of reported cases. The top 10 countries in terms of cases cases were chosen from among the UE+EFTA+UK. Predictions are the number of cases at April 30, May 2, and May 4, respectively; lower and upper bounds can be seen inside brackets. In bold reported cases that were inside prediction intervals. K is the predicted final number of cases.

Countries	Cases	April 30		May 2	
		Prediction	Reported	Prediction	Reported
Spain	213942	215365 [213942-220067]	**215183**	217465 [213942-222263]	**217804**
Italy	201505	203403 [201505-206418]	**203591**	206856 [203761-209951]	**207428**
United Kingdom	161145	165268 [162977-167560]	**165221**	172953 [170582 -175324]	177454
Germany	157641	158753 [157641-160596]	**159119**	160718 [158837-162598]	**161703**
France	126835	127941 [126835-130010]	**128442**	129882 [127761-132004]	**130185**
Belgium	49227	49698 [49227-51501]	**49741**	50616 [49227-52459]	**50565**
Netherlands	38416	38889 [38416-41086]	**38802**	39462 [38416-41696]	**39791**
Switzerland	29181	29279 [29181-29545]	**29324**	29433 [29181-29703]	**29622**
Portugal	24324	24654 [24324-25941]	**24692**	25113 [24324-26427]	**25351**
Ireland	19877	20253 [19877-21523]	**20253**	20791 [19877-22087]	**20833**
Countries	Cases	May 4		Parameter K	
		Prediction	Reported		
Spain	213942	219240 [214283-224197]	**219205**	239508	
Italy	201505	209946 [206707-213185]	**210717**	232434	
United Kingdom	161145	180122 [177596-182648]	186599	318644	
Germany	157641	162370 [160428-164311]	**163175**	194071	
France	126835	131599 [129385-133812]	**131287**	163290	
Belgium	49227	51399 [49488-53309]	**50990**	67095	
Netherlands	38416	39918 [38416-42206]	**40571**	52600	
Switzerland	29181	29554 [29278-29830]	**29822**	32352	
Portugal	24324	25497 [24324-26855]	**25524**	28376	
Ireland	19877	21232 [19896-22569]	**21506**	39108	

We fit the function over time to be able to predict the evolution of the cumulative cases to generate some useful information which may help political institutions to adopt appropriate control measures; see supplementary S1 Fig for approximations of a selection of countries in Europe. Such curves are based on the calculation of the values of *a*; see supplementary S2 Fig, and *K*, see supplementary S3 Fig, in the selection of countries.

### Evaluation of the errors in the short-term predictions obtained with the Gompertz model

To evaluate the quality of the predictions we systematically ran the prediction routines along the past, for all the days of the spreading of Covid-19 in all countries with more than 1000 cases as of April 11, 2020. We compared the prediction with the actual number of cases to give rise to two different indexes: first, the average relative error of the prediction with the real quantity, and, second, the determination of whether the real quantity was within the error of the prediction. These two indexes allow us to calibrate the error bars of the model since we can calculate the percentage of success.

To construct the predictions we used all the data available from the day where cumulative cases crossed the threshold value of 100 cases. However, the successive changes in the control measures could affect the parametrization of the curves. We improved the predictions employing only the last 15 values of the data, after the start of local transmission in the epidemics.

**Fig 6:**
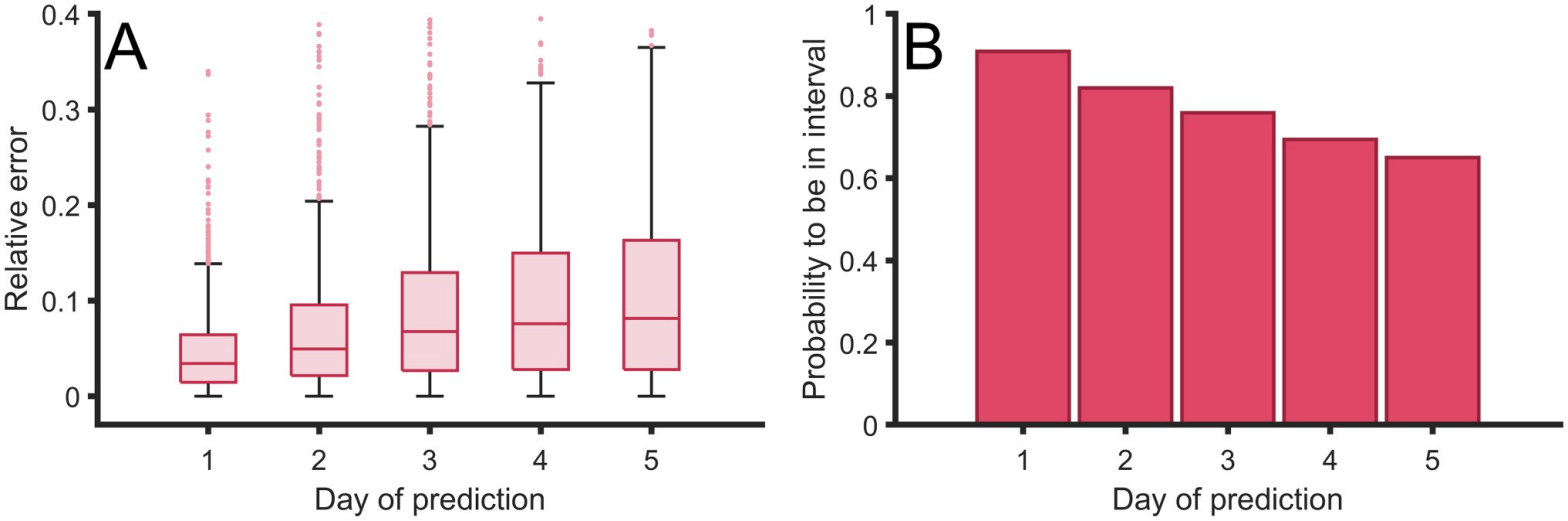
Error of the predictions done by the dynamical fitting of Gompertz function. (A) Relative error between the predictions of the confirmed case for the next five days, in comparison with the actual confirmed cases in several countries. (B) Probability of obtaining the actual real value within the interval of confidance inside the error bars for the next five days. Errors computed with retrospective using all countries with over 1000 cases on April 9, 2020 using ECDC reported cases [28].

In Fig 6 we show the relative error of the predictions with respect to real data. First, we obtain relative errors for the prediction for the next day of around 2%. The error increases for the predictions for the next days up to the average error of around 5% for the fifth day; see Fig 6A.

The predictions are obtained with a certain error due to uncertainty in the estimation of the parameters of the Gompertz function. Therefore, we evaluated, in Fig 6B, the probability of the actual value being within the prediction intervals around the predicted value. The probability for the first day is around 90% of confidence while this probability decreases for the next days to around 60% for the fifth day; see Fig 6B. We certainly were successful in predictions at short-times of the cumulative cases and therefore the new cases, and, as expected, the accuracy of the predictions decayed over time.

### Short-term prediction error is corrected with filters

For the predictions made in the previous section on a given day, we used the reported data from 15 days before in order to fit the parameters of the Gompertz function, giving the same weight to all 15 days. From the methodological point of view we improved our predictions using filters to give more relevance to the last data points. We were able to give more weight to the last days and compare with the prediction considering all data points with the same standing. This may be especially useful to rapidly capture changes in trends, as for instance those that we found around the peak of new cases.

**Fig 7:**
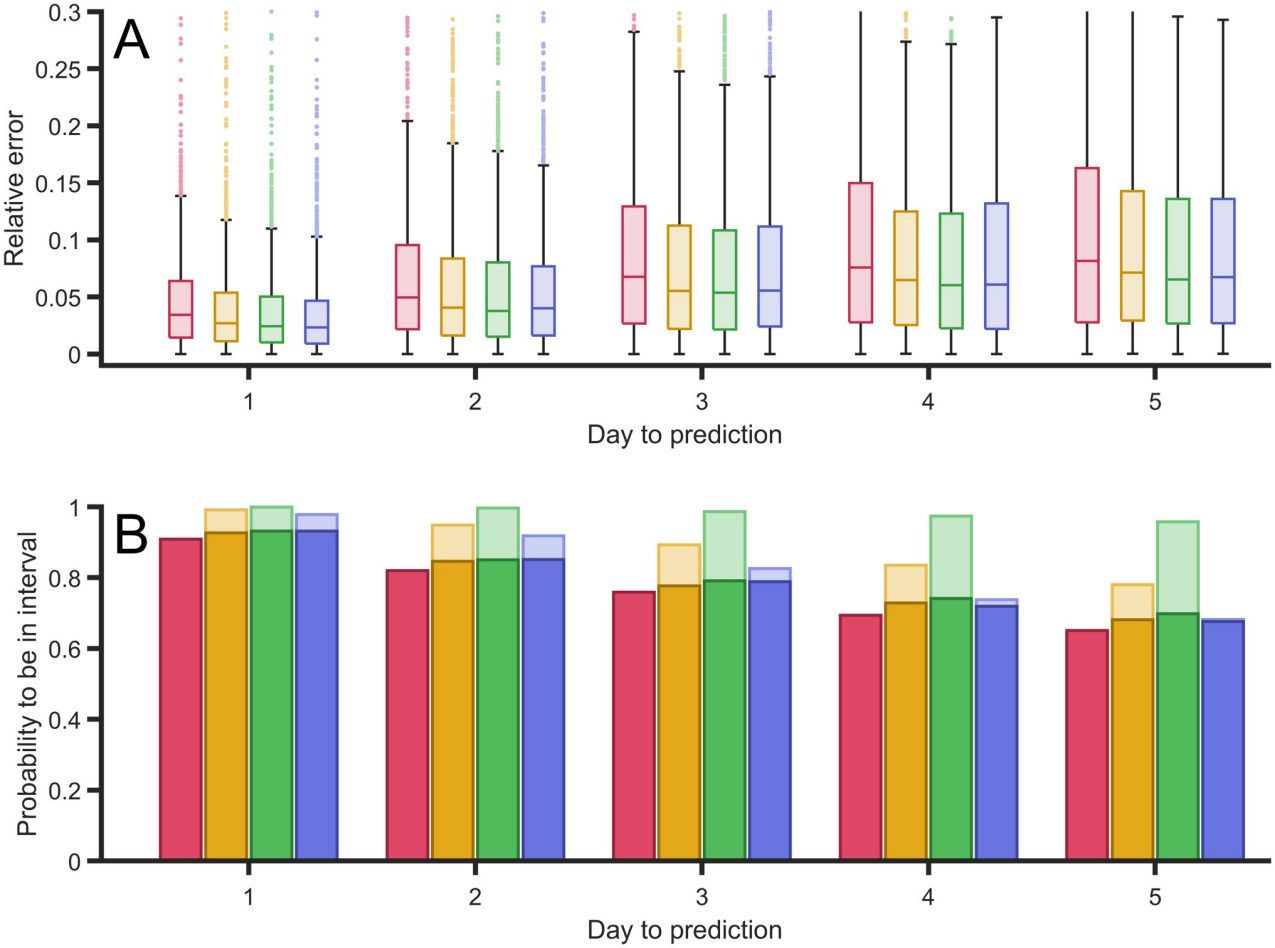
Error of the predictions with different type of filters. (A) Relative error between the predictions of the confirmed case for the next five days, in comparison with the actual confirmed cases for four different types of filters: constant 15 values (red), linear increase (orange), parabolic increase (green), and a filter with three largest last values (blue). (B) Probability of obtaining the actual real value within the interval of confidance inside the error bars for the next five days using the same four filters. Light bars to the probability of being found within the confidence interval using each filter confidance interval;, dark bars show probability of being within the confidence interval using first filter confidance interval to be able to compare among the different filters. Although different filters have different confidence intervalsizes, they have the same significance level of *α* = 0.01.

We tried several options and concluded that three different filters must be analyzed. We proceeded to show how they behave using the data sets for different countries available. The first filter consists of linear increase in weight between the first and the fifteenth day, the second one a parabolic growth of the weight, and, finally, the third one gives more relevance to only the last three days (a hundred times larger than the other twelve days). By comparison with the equal weight and the other three filters, we obtained a filter which minimizes the relative error; see the comparison in Fig 7A.

Although comparison among the four procedures, see Fig 7, shows relatively small differences, this statistical study shows performs better with the last filter, which gives greater weight to the last three values of the data. The performance of such filter is particularly better when the epidemic approaches the values of the peak of new cases.

The average of the relative error decreases with the asymmetry of the type of filter we employ; see Fig 7A. The filter with greatest weights in the last three events presents a better performance in comparison with the other three filters employed. It is important to note that we also checked other filters with greater weight in the last single event and the last two events, and the results were less accurate.

We obtain similar results if we evaluate the probability of success of the predictions of each filter; see Fig 7B. Light bars in such a figure show success using the error bars obtained from the mean square method adapted to each of the filters. Note that the error bars, or confidence intervals, of each method may be different and therefore this may affect the likelihood of success because it produces larger confidence intervals. To systematically compare the four methods we employed the confidence interval of the original method with the mean values obtained in the other filters. Note that with such definition, the dark and light bars for the first method overlap. We also observed better performance in increasing the asymmetry of the filter and as in the previous comparison, the method focused on the last three values maximized the probability of success.

## Discussion

Finally, we discuss the possibility of longer-term estimations with the Gompertz function, and offer our main conclusions.

### Long-term estimations can be obtained from Gompertz model

We are assuming simple premises and they permit us to expand the short-term predictions shown above and calculate longer-term predictions greater than five days, explicitly the values of *K*, *t*_*p*_ and 90%*K*. Long-term estimations are possible during a certain outbreak wave. Second waves may completely change the dynamics and the values of final incidence. Such new epidemic focuses are not considered in the model, we may treat them as an independent epidemic for which the numbers probably have to be reset.

The use of a phenomenological function facilitates the projection to the future of the trend in comparison with other methods which evaluate in the vicinity of the last day. Although the only relatively reliable predictions in such a complicated problem are short-term predictions, we can however address relevant questions like the final value of total cases of parameter *K*, predictions of the peak or maximum of new cases, or the time needed to arrive at 90% of the total cases. To obtain such long-term estimations we employ the whole data set for each country to unveil the trend of the whole dynamics.

**Fig 8:**
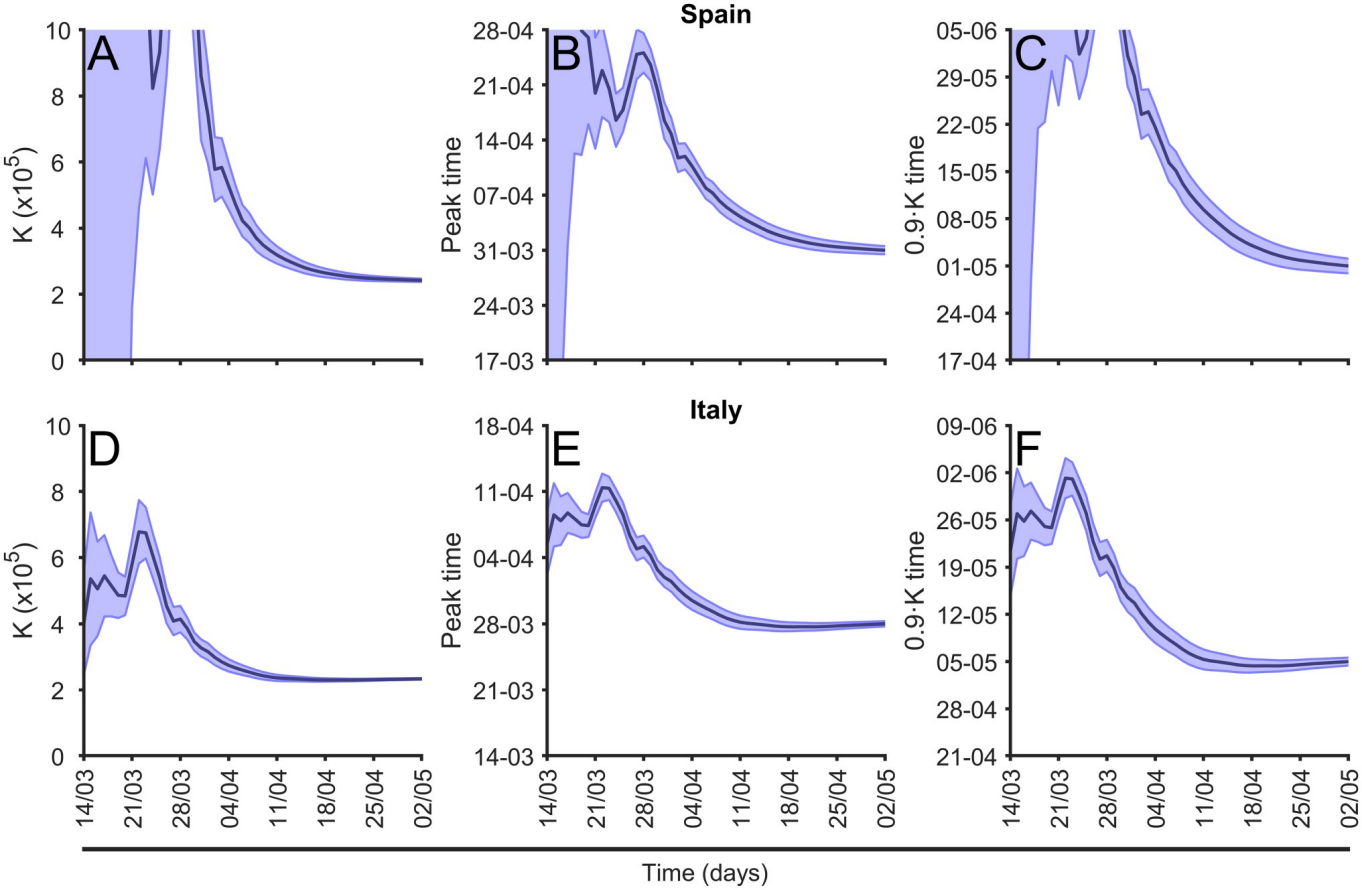
Evolution of the long-term estimations. (A) and (D) Evolution of the prediction of final total number of cases, *K*; (B) and (E) evolution of prediction of the time for the peak of maximum new cases prediction, see Eq (6) and (C) and (F) evolution of the arrival to 90% of total cases between March 14, 2020 and May 2, 2020 in Spain and Italy, respectively.

We calculate daily the parameter values of the fitting function described above and the evolution of the parameter *K* for different countries together with two characteristic times of the epidemic. See two examples, Spain and Italy, in Fig 8, for the value of *K*, *t*_*p*_, and 90%*K*. For other countries in Europe see, respectively, supplementary S3 Fig, supplementary S4 Fig, and supplementary S5 Fig. The estimations begin with large uncertainty; however, the values converge on the actual value systematically for the three calculations. The confidence interval also reduces with time, although there are systematic fonts of errors not addressed by the interval. The main differences between Spain and Italy in Fig 8 are the large errorbars for Spain at the beginning of the evolution, because of the delay in the epidemic phase of both countries on March 9th, when the graphic begins. While in Italy the epidemic was fully developed, in Spain the epidemic was at the initial phase with an exponential growth.

**Fig 9:**
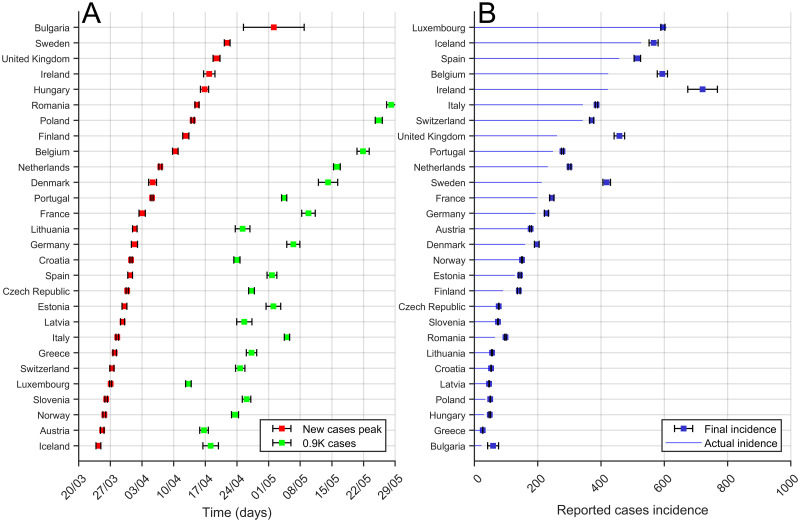
Comparison of long-term estimations among European Countries. (A) Time for the peak prediction (red) and the time to arrive at 90% of total cases (in green) predictions obtained from the last evaluation of the Gompertz function (April 12, 2020) to the evolution of the cumulative cases. Countries are sorted from top to bottom using time-to–peak-time prediction. (B) Final incidence (total cases per 10^5^ inhabitants) prediction (blue squares) obtained from the last evaluation of the Gompertz function (April 12, 2020) to the evolution of the cumulative cases (blue line); see procedure in Fig 8. Error bars correspond to the error obtained from the fit and the corresponding error propagation. Countries are sorted from top to bottom in terms of actual incidence.

Using the method described above we can compare the three predictions shown in Fig 8 for all the countries in Europe for a particular date; see this comparison in Fig 9. For the two temporal comparisons note that actually the dates for the peak for some countries had already been passed at the time when the evaluation was made. However, it is actually not always clear when the actual moment a country is passing the peak is. Furthermore, for the comparison among the different countries in Europe with very different demographics, we used the incidence of the epidemic, evaluated as the number of cases per 10^5^ inhabitants. In this graphic we compare with the actual phase of the epidemic at May 2, 2020 in each country [22]. While some of the countries are close to the final number of cases, there are some countries still at the initial phase of the epidemic with very large growth, which predicts large incidence rates. This is the case with the United Kingdom.

Note that we have to approach the previous estimations reticently, because they are only approximations assuming some simple premises. Therefore, we consider such estimations as objects for discussion rather than as results of the model.

### Outlook and conclusions

We fitted the Gompertz function to the cumulative cases in different regions and countries to be able to infer, from the fitted parameters of the model, relevant quantities for the understanding of the epidemics. On the one hand, we obtained reliable short-time predictions for the new cases during the subsequent days. These predictions are robust and the percentage of success is around 90% for the next day. but in addition, the fitting provides some long-term quantities, for example, estimations of the total number of cases or the timing of the peak of new cases.

As an empirical function, Gompertz does not depend on previous knowledge of the system. It is especially useful in situations where there is no deep knowledge of the internal structure of the epidemics and when key properties of the epidemics are not known. It is precisely the lack of knowledge regarding the different pathways of contagion or its dependence on social measure that makes the fitting of a quantitative predictable model impossible. Complex models with a lot of parameters to fit are, in this type of epidemic, exercises in exploring possible scenarios, but never real quantitative tools. No model can predict the reaction of the population to a particular measure, nor even properly assess the parameters of mobility when even basic immunity questions remain unsolved. This is what makes our results about the large degree of confidence in terms of short-term predictions of the evolution of the Covid-19 epidemics so important. Our work has important ramifications since it can predict, and at the same time assess, changes in the dynamics of the pandemic. The prediction procedure adapts to changes in any of the structural properties of the system. Changes in the diagnostic testing needed to detect a case, in social measures, or in the way of counting cases just introduce variation in the model that fades away as the new properties emerge again. We have clearly shown in this paper that this changing structure is properly captured with the decreasing nature of the growth given by parameters *μ*_0_ and *a*, and the final number of cases *K*. The highly complex and unknown nature of key elements of the epidemics does not prevent our predicting its evolution in the short-term and to assess the control, or lack thereof, of the epidemics’ spread.

We may conclude that the methodology here presented can be further employed for the evaluation of the epidemic and the control measures in the next countries to which it spreads in its initial stage. We obtain predictions with a success greater than 90%, which means that around 90% of the reported cases are within the prediction intervals.

We are planning to further collaborate with health institutions in Africa and America to advise them with the predictions of the model for the evolution of the Covid-19 epidemic in these countries. In such collaboration, the continuous interplay between predictions and results during spreading will lead us to a rethinking of the assumptions of our model. We hope to further improve the predictions by the introduction of changes, if needed. Further work can be done to improve the prediction process. The results of the fitting might be better if country-wide data is disaggregated for more homogeneous subnational regions. Data shows that in some countries the appearance of different focuses produces the formation of different epidemics which under the conditions of strong restriction of movement can give rise to independent dynamics within the country. It is more reliable to work with information at the regional level although the number of cases is lower and the fluctuations stronger. We have observed good statistical behavior and predictions in the secondary outbreak in several Catalan cities [36] with a total population of around half a million or more. We expect that models applied to regions of this size could be useful to predict more aggregated scales. However, the main limitation of the regional approach up to now has been the lack of detailed data and/or the difference in the protocols and definitions used by local authorities. As the pandemic advanced more reliable data at the regional level was available; see for example [37].

Finally, we would like to note that the use a generic function is an empirical tool to treat future local and global epidemics, as has been begun recently with other growth functions like the Verhust and Richards models [17]. We plan to continuously update the approach employed here to adapt to any special particularity of any new epidemics. Presently, the same data are applied to guide public policy in hospital administrations giving assessment to regional governments regarding the short-term evolution of health needs.

In order to take adequate and precise control measures political leaders need up-to-date information on the epidemics and a clear representation of the phase of the epidemics among several countries or in a particular country of different regions. We have found our short-time predictions to be a highly valuable information tool for policymakers, since they it can help guide their short-term planning decisions.

## Supporting information

S1 Fig Cases in different European countries. The total cases together with the new daily cases with the corresponding fitings obtained from the Gompertz model are shown for a selection of European countries.
(TIF)

S2 Fig Evolution of the fitting of parameter *a*. The dynamics of the fitting of parameter *a* obtained from fitting from the Gompertz model are shown for a selection of European countries.
(TIF)

S3 Fig Evolution of the fitting of parameter *K*. The dynamics of the fitting of parameter *K* obtained from fitting from the Gompertz model are shown for a selection of European countries.
(TIF)

S4 Fig Evolution of the fitting of parameter *t*_*p*_. The dynamics of the fitting of parameter *t*_*p*_ obtained from fitting from the Gompertz model are shown for a selection of European countries.
(TIF)

S5 Fig Evolution of the fitting of parameter 90%*K*. The dynamics of the fitting of the parameter 90%*K* obtained from fitting from the Gompertz model are shown for a selection of European countries.
(TIF)
