# Supplementary figures and images for: Empirical model for short-time prediction of COVID-19 spreading
Source: PLoS Comput Biol. 2020 Dec 9;16(12):e1008431. doi: 10.1371/journal.pcbi.1008431 (PMC7725384; doi:10.1371/journal.pcbi.1008431)

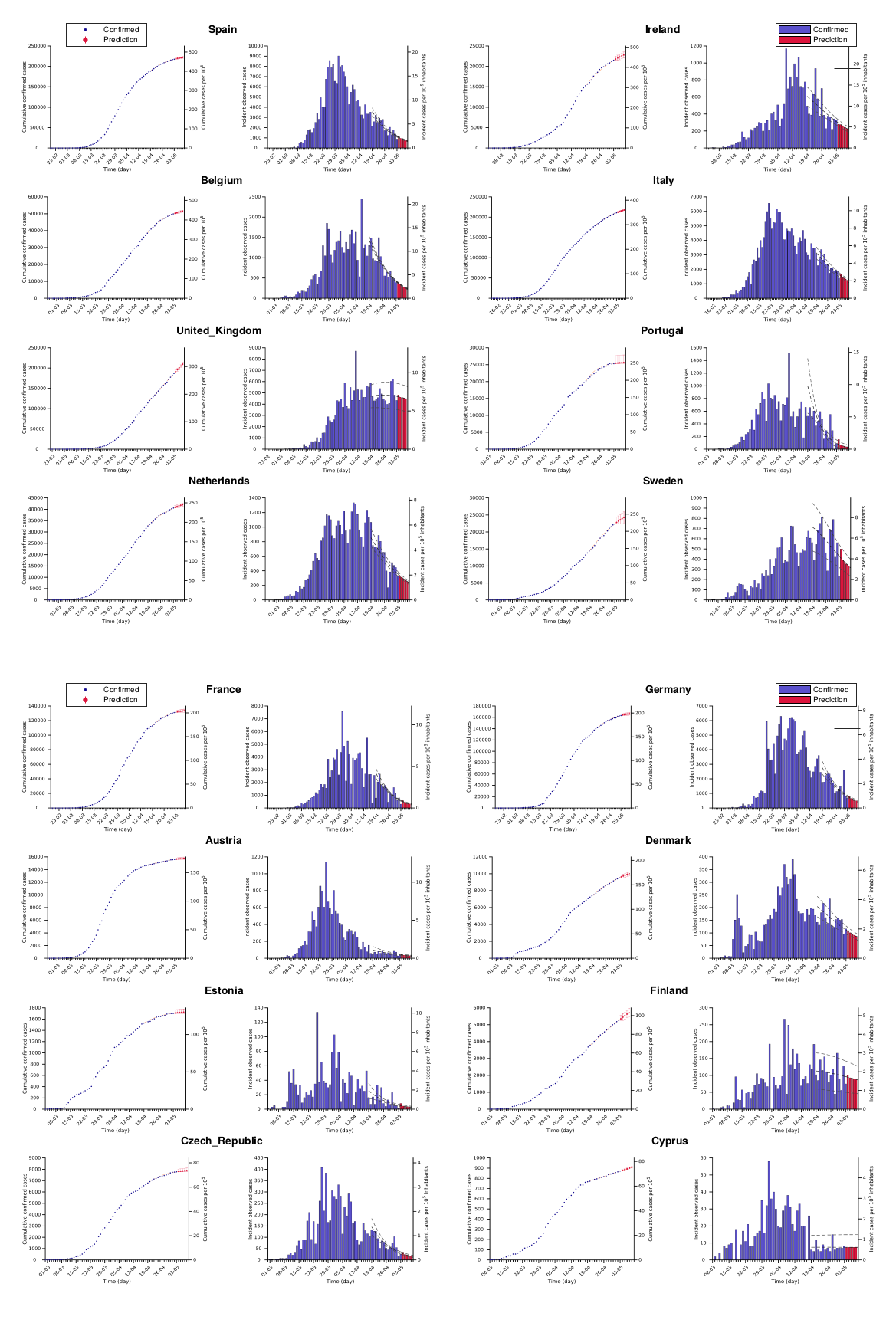

Supplement: S1 Fig — The total cases together with the new daily cases with the corresponding fitings obtained from the Gompertz model are shown for a selection of European countries. (TIF) [file pcbi.1008431.s001.tif]

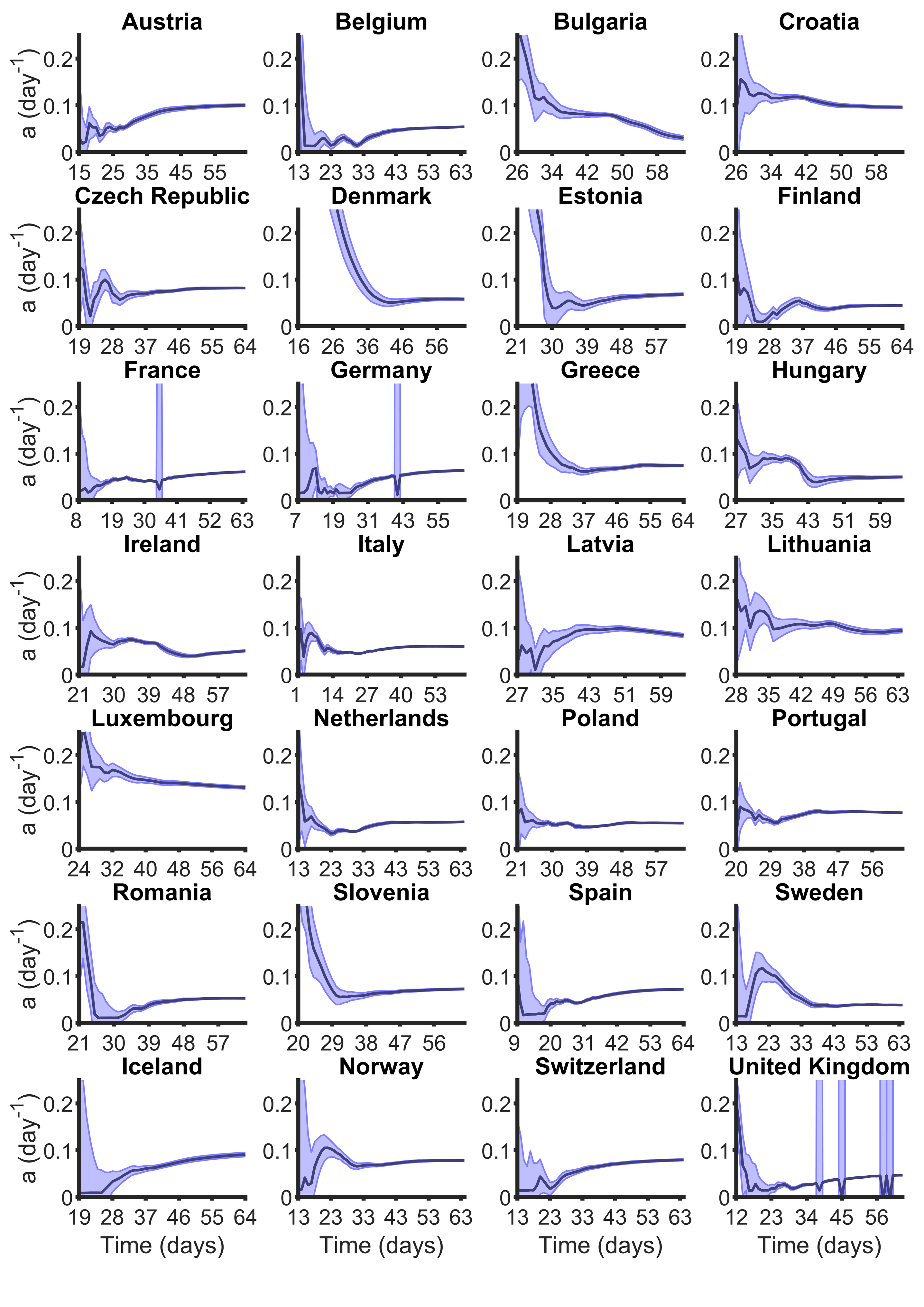

Supplement: S2 Fig — The dynamics of the fitting of parameter a obtained from fitting from the Gompertz model are shown for a selection of European countries. (TIF) [file pcbi.1008431.s002.tif]

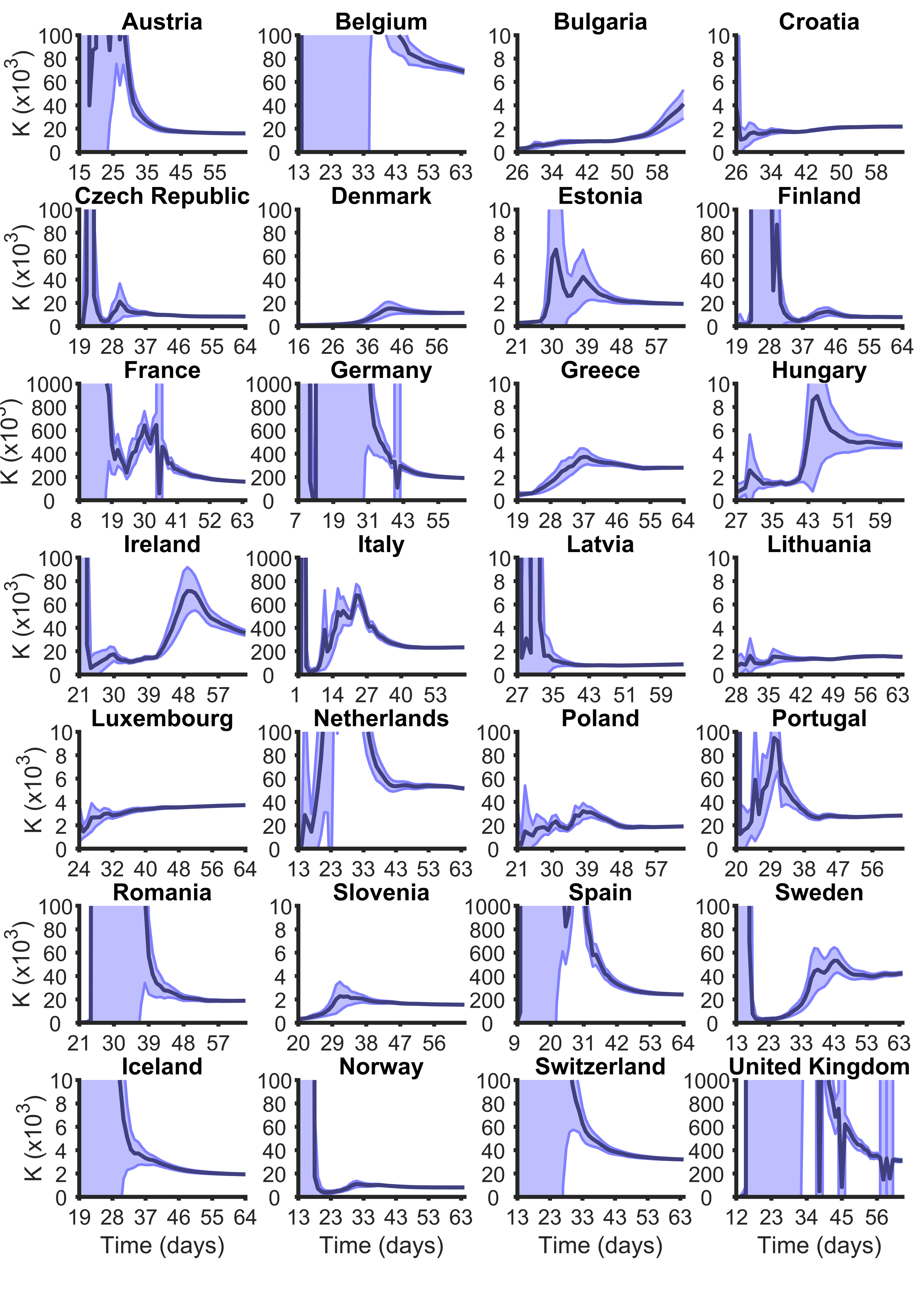

Supplement: S3 Fig — The dynamics of the fitting of parameter K obtained from fitting from the Gompertz model are shown for a selection of European countries. (TIF) [file pcbi.1008431.s003.tif]

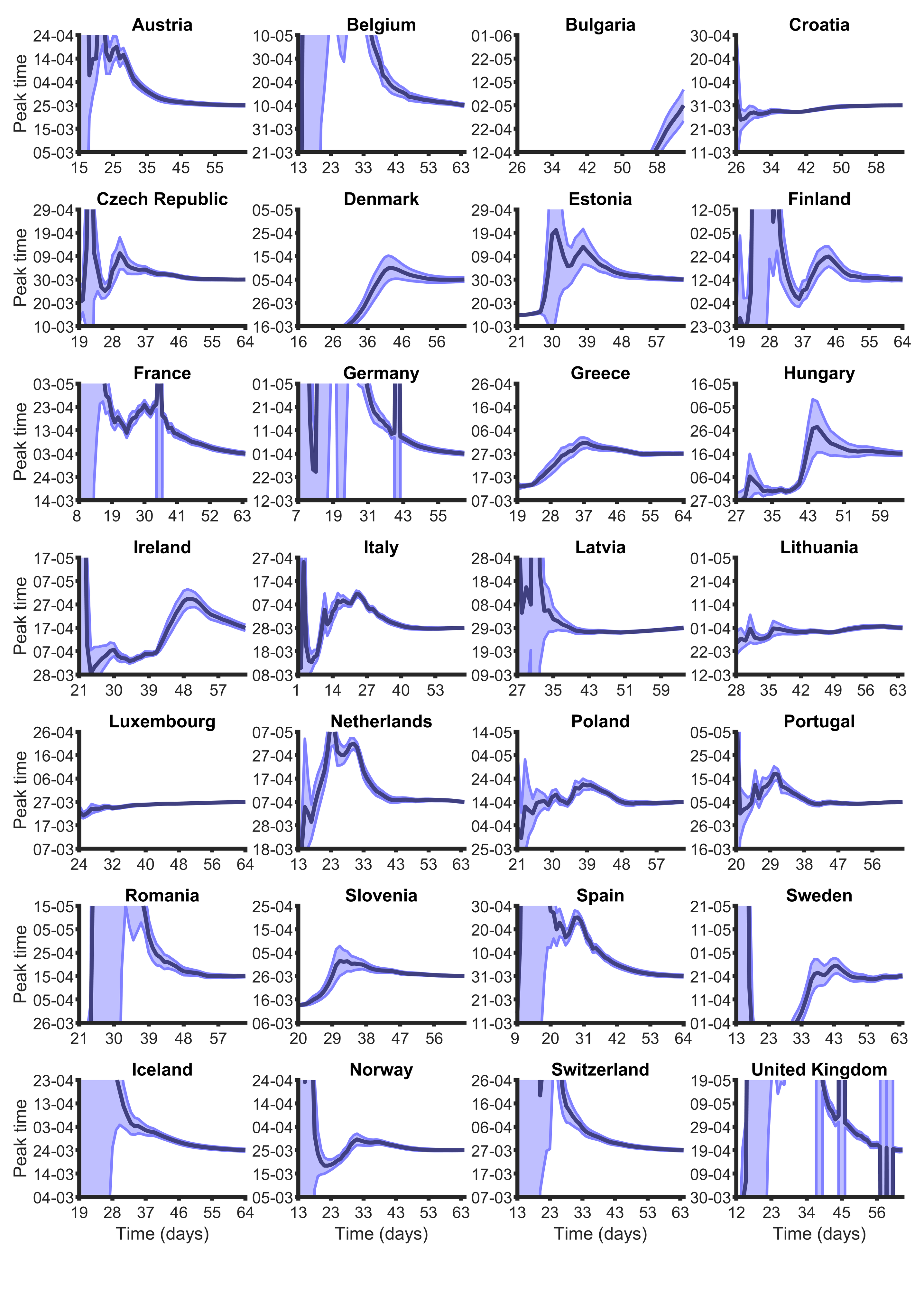

Supplement: S4 Fig — The dynamics of the fitting of parameter tp obtained from fitting from the Gompertz model are shown for a selection of European countries. (TIF) [file pcbi.1008431.s004.tif]

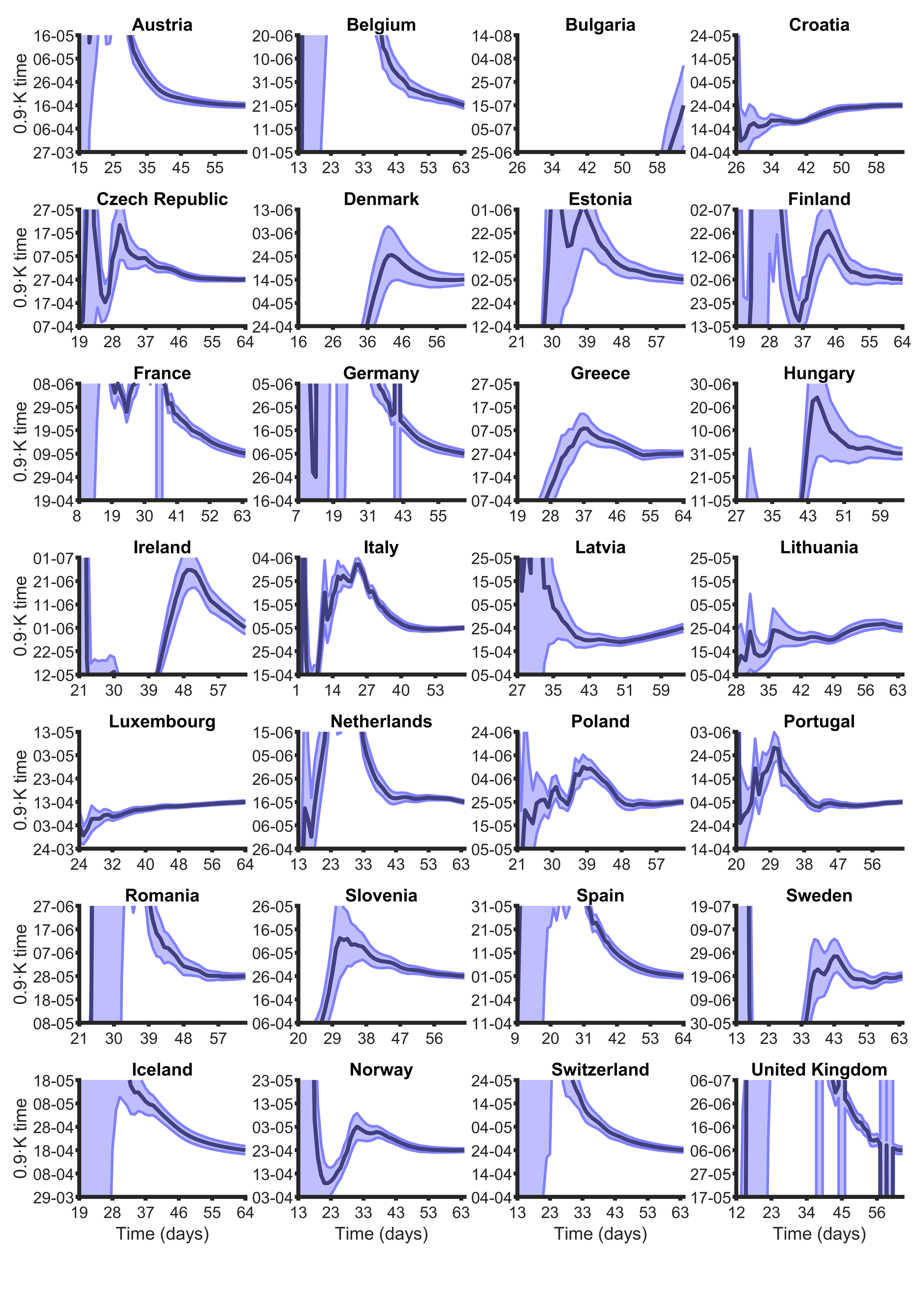

Supplement: S5 Fig — The dynamics of the fitting of the parameter 90%K obtained from fitting from the Gompertz model are shown for a selection of European countries. (TIF) [file pcbi.1008431.s005.tif]
